# Targeted nutritional prehabilitation for high-risk Crohn’s disease patients undergoing elective gastrointestinal surgery: a case series

**DOI:** 10.3389/fnut.2026.1731678

**Published:** 2026-03-03

**Authors:** Francesca Vincenzi, Federica Gaiani, Maria Clotilde Carra, Nicola de’Angelis

**Affiliations:** 1 Unit of Gastroenterology and Endoscopy, University Hospital of Parma, Parma, Italy; 2 Department of Medicine and Surgery, University of Parma, Parma, Italy; 3 Department of Translational Medicine and LTTA Centre, University of Ferrara, Ferrara, Italy; 4 Department of General Surgery, Fondazione Poliambulanza, Brescia, Italy

**Keywords:** Crohn’s disease, Crohn’s disease exclusion diet, partial enteralnutrition, postoperative outcomes, preoperative nutrition

## Abstract

**Background:**

Crohn’s disease (CD) is a chronic inflammatory bowel condition often leading to complications requiring surgery. Optimizing nutrition before surgery contributes to reducing postoperative complications. The Crohn’s Disease Exclusion Diet (CDED), combined with Modulen as partial enteral nutrition (PEN), has been shown to help restore the intestinal barrier, promote a balanced immune response, and mitigate the inflammatory process (Modulen: Nestlé Italia S.p.A: Milan, Italy). Thus, this approach may be implemented into a prehabilitation protocol to tailor nutritional interventions for candidates undergoing CD surgery.

**Methods:**

We describe four adult CD patients who underwent preoperative nutritional optimization with CDED+PEN. Interventions lasted 2–12 weeks, providing 30–35 kcal/kg/day, and were tailored based on weight and appetite. Adherence, nutritional markers, medical therapy, and surgical outcomes were monitored.

**Results:**

All patients showed good adherence and tolerance to CDED+PEN, with no flare-ups or treatment discontinuations. CDAI decreased in three of four cases. Nutritional status was maintained in all cases. CRP and albumin remained within normal values. Two patients discontinued biologics before surgery without complications, while two were managed without pharmacological therapy. All patients underwent robotic intestinal resection with no postoperative complications.

**Conclusion:**

CDED+PEN may be incorporated into the prehabilitation protocol to enhance the nutritional and inflammatory status of CD patients undergoing surgery. Clinical trials assessing its efficacy and tolerance are warranted.

## Introduction

Crohn’s disease (CD) is a chronic, relapsing–remitting inflammatory bowel disease (IBD) characterized by transmural inflammation that can affect the entire intestine and particularly the distal ileum (1). The pathogenesis of CD is multifactorial, with the well-established role of inflammatory cells in maintaining disease activity (2). Complications include stricturing or penetrating forms, affecting approximately 70% of CD patients and often requiring elective surgery within 20 years of diagnosis. Malnutrition affects 65–75% of CD patients (1).

CD treatment is multidisciplinary, and its principal aims are to reduce active inflammation and to achieve and maintain clinical remission. Dietary therapy has been increasingly recognized as a key management strategy for CD, including the use of exclusive enteral nutrition (EEN) as a first-line therapy for pediatric CD, according to current guidelines (3). Although EEN has been demonstrated to be more effective than corticosteroids in inducing CD clinical remission with no medical side effects (4, 5), its use could be limited by poor adherence, particularly in adult patients (1).

The Crohn’s Disease Exclusion Diet (CDED) is a novel dietary therapy specifically designed for patients with CD to limit certain nutritional components, thereby reducing inflammation and achieving remission (4, 6). The CDED consists of three phases (phase 1, weeks 1–6; phase 2, weeks 7–12; and phase 3, from week 13). In phase 1, 50% of calories are provided via partial enteral nutrition (PEN), and the remaining 50% come from a diet low in fiber, taurine, and saturated fat (4). The primary intention of this exclusion diet is to help restore the intestinal barrier, promote a balanced immune response, mitigate the inflammatory process, and facilitate the healing of the mucosa (7). Studies have shown that CDED is associated with a significant and progressive reduction in CRP and fecal calprotectin, which are objective markers of intestinal inflammation (6, 8). Incorporating regular food meals into the diet also improves tolerance of the dietary regimen (8).

Nutritional support is also critical in the surgical setting. Poor nutritional status, including a body weight loss of more than 10% in the 6 months preceding surgery, has been associated with worse postoperative outcomes (1, 9). Accordingly, the latest guidelines from the European Society of Clinical Nutrition and Metabolism (ESPEN) (10, 11) and the European Crohn’s and Colitis Organization (ECCO) (12, 13) recommend assessment of nutritional status in all patients scheduled to undergo surgery and implementation of dietary interventions in those who are malnourished or at risk of malnutrition.

EEN is indicated in the preoperative period for patients with stenosing or fistulizing disease, aiming to improve nutritional status and reduce postoperative complications (14). Presurgical EEN has been shown to increase median serum albumin levels and significantly decrease C-reactive protein (CRP) (15, 16). By reducing CD-related inflammation, EEN may also allow a decrease in pre-operative steroid use and optimize patients’ fitness for surgery (17). Despite this evidence, EEN can be challenging to implement in surgical patients due to limitations such as poor palatability and difficulty adhering to a liquid diet for an extended period. Non-adherence to the treatment has contributed to the limited success of EEN in many studies and in clinical practices (1). Conversely, CDED has been shown to have comparable efficacy in terms of remission and reduction of pro-inflammatory markers but better compliance than EEN. Thus, it may also be a suitable approach for patients undergoing surgery.

To date, evidence on CDED use in the preoperative setting is very scarce. To our knowledge, the only available clinical study was published by Wall et al. (18). This New Zealand feasibility study evaluated CDED versus EEN versus standard care for patients who were not malnourished (18). The study, conducted on 17 patients, demonstrated a low rate of postoperative complications in all treatment groups. CDED was well tolerated, with no exacerbation of gastrointestinal symptoms or significant weight change. In the CDED group, four of five (80%) patients reported high adherence, with more than 90% of their total energy intake from CDED. In comparison, EEN treatment was less tolerated and resulted in lower treatment retention, with four of six patients withdrawing or changing treatment (19).

This perspective article describes the cases of four adult CD patients who received CDED + PEN in the preoperative period as part of a prehabilitation protocol aimed at improving patients’ fitness for surgery and postoperative outcomes. The enrolled patients were the first four consecutive patients observed. Our patients were initiated on and educated about the dietary treatment by a dietitian, who then assessed adherence and tolerability. The study is reported in accordance with the CAse REport (CARE) Guidelines and Checklist to ensure transparent and high-quality reporting (19). Patients’ clinical information is reported, ensuring respect for their privacy. All patients were treated with dignity and protected from any possible harm. The main clinical information is summarized in Table 1.

**Table 1:** Baseline clinical features.

Clinical characteristics	Case 1	Case 2	Case 3	Case 4
Sex	F	F	F	M
Age at diagnosis	43	31	29	30
Age at surgery	46	58	29	50
Previous surgery	None	Yes	None	Yes
Therapy before surgery	Anti-TNF alfa Ustekinumab	Anti-TNF alfa Risankizumab	None	Azathioprine
Therapy at surgery	Anti-TNF alfa Ustekinumab	Risankizumab	None	None
Phenotype disease*	A3, L3-L4, B1	A3, L3, B2	A2, L3, B2	A3, L3, B2
Endoscopic activity (SES-CD) before surgery**	16	16	15	14

*Phenotype disease: Montreal Classification. **Simple Endoscopic score for Crohn’s Disease (SES-CD).

## Case reports

### Case 1

We present a case of a 43-year-old Bangladeshi woman who presented for approximately 1 year with abdominal pain and diarrhea associated with low-grade fever and approximately 15% weight loss since symptom onset. Colonoscopy revealed pancolitis with serpiginous ulcers in the ileum (Simple Endoscopic Score for Crohn’s Disease - SES-CD 36). Esophagogastroduodenoscopy (EGDS) showed gastric antral ulcers and ulcers in the duodenal bulb (Montreal Classification A3, L3-L4, B1). A diagnosis of CD was made and confirmed by histological examination. The patient was initially treated with prednisone (50 mg/day for 4 weeks, then gradually tapered), and due to persistent endoscopic disease activity, infliximab was subsequently administered at 5 mg/kg every 8 weeks, later increased to 10 mg/kg every 4 weeks. After a prolonged period of clinical remission, the patient was compelled to reduce infliximab to 7.5 mg/kg at 4-week intervals due to the development of lesions consistent with a paradoxical psoriatic manifestation. After 5 months, an increase in inflammatory indices was observed: CRP levels increased to 10 times the normal value, and calprotectin levels reached 450 μg/g (normal value [n.v.] < 50 μg/g), without any other complications. Anti-IL12/23 therapy (ustekinumab) was therefore added to infliximab; however, no reduction in inflammatory indices was noted. During colonoscopy, an inflammatory stenosis of the ascending colon/ileocecal valve (SES-CD 16) was detected, without septic complications. The patient was referred to the surgical evaluation.

Four weeks before surgery, ongoing biological therapies (anti-TNF alpha and anti-IL 12/23) were suspended, and phase 1 CDED with PEN using Modulen was initiated, providing a total of 2,000 kcal/day (of which 1,000 kcal/day was from Modulen). Follow-ups every 2 weeks showed good adherence and tolerability to the diet. After 4 weeks, the Crohn’s Disease Activity Index (CDAI) decreased from 125 to 111, and no significant weight change was observed. Blood tests were within the normal range, except for a slight increase in CRP levels (Table 2).

**Table 2:** Summary of patient 1’s pre-surgical tests.

Parameter	Baseline	Week 4
CDAI	124	111
Body weight	58 kg	59 kg
BMI	21.8	22.2
BIA	Normal body composition	
Albumin	3.9 g/dL	4 g/dL
Pre-albumin	26 mg/dL	30 mg/dL
CRP	50 mg/dL	10 mg/dL (n.v. 5 mg/dL)
Fecal calprotectin	>1,000 μg/g	NA

CDAI, Crohn’s Disease Activity Index; BIA, Body Impedance Assessment; BMI, body mass index; CRP, C-reactive protein; NA, not available.

### Case 2

We present the second case of a 58-year-old people of European descent woman who reported a recurrence of diarrhea lasting approximately 1 year, without blood or weight loss. She has had CD since the age of 31 years and was previously treated with emergency ileal resection surgery for an enterovesical fistula in 2001. Stool testing revealed high levels of fecal calprotectin (300 μg/g). Colonoscopy showed an inflammatory substenosis of the surgical anastomosis (Montreal Classification A3, L3, B2; SES-CD 12), confirmed by histological examination. Entero-MRI confirmed thickening of the perianastomotic ileal loop, showing mild dilation upstream of the anastomosis, with lengthening and approximation to the bladder. Anti-TNFα therapy was initiated with adalimumab 40 mg administered subcutaneously every 2 weeks and subsequently optimized to a weekly schedule. Following a temporary clinical benefit, the increase in calprotectin (350 μg/g) prompted a change in biological therapy to anti-IL-23 (risankizumab). After 6 months, colonoscopy revealed a tight stenosis of the surgical anastomosis (SES-CD 16). Entero-MRI confirmed the localization of the disease with a sub-stenosing fibrotic, characterized by thickening of the pre-anastomotic ileal tract extending approximately 6 cm, along with significant dilation of the upstream ileal tract. Following an episode of sub-occlusion, surgical resection of the stenotic tract was scheduled. A preoperative CT scan excluded the presence of abscess complications.

In anticipation of surgery, ongoing biological therapy was suspended, and phase 1 CDED with PEN using Modulen was initiated, providing a total of 2,000 kcal/day (of which 1,000 kcal/day was from Modulen). Follow-ups every 2 weeks showed good adherence and tolerability to the diet. After 6 weeks, the CDAI decreased from 119 to 106. Evaluated inflammatory markers were within the normal range (Table 3). No significant weight change was observed.

**Table 3:** Summary of patient 2’s pre-surgical tests.

Parameter	Baseline	Week 6
CDAI	119	106
Body weight	62 kg	62 kg
BMI	23.3	23.3
BIA	Normal body composition	NA
Albumin	3.9 g/dL	3.9 g/dL
Pre-albumin	19	27 mg/dL
CRP	6.4 mg/dL	Normal value
Fecal calprotectin	233 μg/g	NA

CDAI, Crohn’s Disease Activity Index; BIA, Body Impedance Assessment; BMI, body mass index; CRP, C-reactive protein; NA, not available.

### Case 3

We present the third case of a 29-year-old Tunisian woman woman with a known diagnosis of diagnosis of stenosing CD of the ascending colon presented to the emergency room with abdominal pain and diarrhea without blood. Colonoscopy revealed severe stenosis of the ascending colon (Montreal Classification A2, L3, B2; SES-CD 15), and histological examination confirmed the diagnosis of CD. Esophagogastroduodenoscopy was negative, and inflammatory markers remained unchanged. An entero-MRI confirmed a diffuse concentric thickening of the cecum and proximal ascending colon, with severe luminal reduction and stenosis over a 6-cm section involving the terminal ileum, with suspected entero-enteric fistulization and without abscess formation.

During the multidisciplinary discussion, surgical treatment was indicated, and phase 1 CDED with PEN using Modulen was initiated, providing a total of 2,000 kcal/day (of which 1,000 Kcal/day was from Modulen), pending surgical planning. A follow-up 2 weeks later showed good adherence and tolerability to the diet. The CDAI and body weight remained stable (Table 4).

**Table 4:** Summary of patient 3’s pre-surgical tests.

Parameter	Baseline	Week 2
CDAI	131	131
Body weight	51 kg	51 kg
BMI	18.5	18.5
BIA	Fat mass at the lower limits	NA
Albumin	3.5 g/dL	3.8 g/dL
Pre-albumin	19 mg/dL	20 mg/dL
CRP	7.5 mg/dL	Normal value
Fecal calprotectin	NA	NA

CDAI, Crohn’s Disease Activity Index; BIA, Body Impedance Assessment; BMI, body mass index; CRP, C-reactive protein; NA, not available.

### Case 4

We present a fourth case of a 50-year-old eople of European descent man diagnosed with CD following emergency ileocecal resection for perforation in 2004, and a subsequent colon resection extending to the transverse colon due to post-surgical complications. The patient had been treated for more than 10 years with low-dose cortisone and sub-therapeutic doses of azathioprine. The patient presented for reassessment of his disease. Abdominal ultrasound showed portal vein thrombosis and splenomegaly, along with thrombocytopenia (72,000/μL). EGDS revealed grade F2 esophageal varices, which were treated accordingly. Entero-MRI confirmed portal system thrombosis and stenosis of the surgical anastomosis, with dilation of the upstream segments and evidence of disease activity over a 30 cm section. Due to the critical presentation, the patient was started on EEN with Modulen (2,500 Kcal/day) and reported subjective improvement in wellbeing. After 4 weeks, the patient began the CDED diet (phase 1, totaling 1,500 kcal/day) with 1,000 kcal/day of Modulen. At the beginning of CDED, the patient had a CDAI of 110, a body weight of 86 kg, and excess fat mass with lean mass depletion, as evaluated by BIA. Follow-ups were conducted every 2 weeks. After approximately 8 weeks of the CDED protocol, colonoscopy revealed a tight stenosis of the ileum/transverse colon anastomosis, with no possibility to perform endoscopic dilatation (Montreal Classification A3, L3, B2, SES-CD 14). Therefore, after excluding hematologic and hepatologic causes underlying the portal vein thrombosis, the patient was deemed a candidate for surgery. During the CDED protocol, the patient reported some difficulty fully adhering to the dietary plan; however, CDAI and body weight remained essentially unchanged (weight loss: 0.05%). Albumin and inflammatory parameters were within normal limits (Table 5).

**Table 5:** Summary of patient 4’s pre-surgical tests.

Parameter	Baseline (Day 0 CDED)	Week 2	Week 8
CDAI	110	110	82
Body weight	86	86	82
BMI	25.5	25.5	24.7
BIA	Excess fat mass, lean mass depletion	NA	NA
Albumin	NA	3.7 g/dL	3.6 g/dL
Pre-albumin	NA	30 mg/dl	35 mg/dL
CRP		7 mg/dL	5 mg/dl
Fecal calprotectin	NA	NA	NA

CDAI, Crohn’s Disease Activity Index; BIA, Body Impedance Assessment; BMI, body mass index; CRP, C-reactive protein; NA, not available.

### Surgery and postoperative outcomes

The four patients underwent robotic intestinal resection surgery performed by the same surgical team using the daVinci surgical system Xi (Intuitive). The procedures and post-surgical outcomes in all cases are summarized in Table 6. The 30-day postoperative period was uneventful.

**Table 6:** Surgical procedure and post-surgical outcomes.

Parameter	Case 1	Case 2	Case 3	Case 4
Surgical procedure	Single-stage lateral ileocecal resection (15 cm ileum) by minimally invasive surgery	Ileocolic resection of the previous single-stage anastomosis	Ileocolic resection with latero-lateral anastomosis by minimally invasive surgery	Resection of the ileocolic stenosis with the creation of a protective stoma due to the criticality of the abdominal picture linked to the presence of portal thrombosis
Complications				
Grade I–IV	None	None	None	None
Hospital length of stay (days)	6	4	6	7
Intra-operative stoma	No	No	No	Planned
Wound infection	No	No	No	No
Other infectious complications	No	No	No	No

## Discussion

In CD management, surgery represents a significant therapeutic component, as more than half of CD patients undergo one or more surgical interventions during their lifetime (12). Prehabilitation, including nutritional assessments and interventions before surgery, is recognized as an effective strategy to optimize surgical outcomes (20). From this perspective, CDED and PEN administered in the pre-surgical period could impact multiple parameters related to postoperative outcomes, including specific markers of nutritional status and inflammation (4, 6, 8), thereby contributing to improved patient recovery.

The four described patients have heterogeneous characteristics; patients 1 and 2 were already on biological therapy and came to surgery due to complications of the disease despite medical treatment. Patient 3 came to surgery at diagnosis with subacute disease and was naïve to biological therapies. In contrast, patient 4 presented with a picture of stenosing CD in a previous surgical anastomosis, complicated by portal hypertension and previous immunosuppressive therapies. At baseline, none of them were diagnosed with malnutrition, defined as body mass index (BMI) < 18.5, according to the WHO definition.

Currently, the therapeutic value of nutritional support during the pre-surgical phase is controversial in patients without a condition of malnutrition. However, the feasibility study by Wall et al., although small in size, supports the rationale for evaluating effective preoperative nutritional optimization regimens to reduce the risk of postoperative complications, even in patients without obvious malnutrition (18). According to this evidence, a recent ECCO consensus supports considering oral nutritional supplementation when EEN is not feasible in people with CD who are awaiting surgery, regardless of their nutritional status (13).

In the described patients, the nutritional intervention lasted a minimum of 2 weeks and a maximum of 12 weeks, including 4 weeks of EEN and 8 weeks of CDED associated with PEN. Nutritional requirements were estimated at 30–35 kcal/kg/day and adjusted throughout the intervention period according to appetite and weight. Adherence to the dietary regimen, assessed by the dietitian every 2 weeks, also via telephone using targeted questions, was good in all patients. ESPEN recommends a minimum period of 7–14 days of nutritional intervention to have a positive impact on postoperative outcomes (11). However, based on individual needs, nutritional optimization may be extended up to 6–8 weeks to prepare the patient better to withstand surgical stress and to improve postoperative outcomes (21). A prolonged nutritional intervention, even up to 3 months, appears to improve body composition and may be a valuable strategy to mitigate the catabolic effects of the surgical stress response and the subsequent negative consequences of lean mass loss in the post-surgical period. The duration of prehabilitation should, however, be personalized to avoid the risk of disease flare-ups or the need for emergency interventions (22).

Regarding concomitant therapies, recommendations for suspending biological therapies before surgery have recently been updated. The 2024 ECCO Guidelines on Therapeutics in Crohn’s Disease: Surgical Management “recommend against cessation of biologics before surgery as the current evidence suggests that preoperative treatment with anti-TNF therapy, vedolizumab, and ustekinumab does not increase the risk of postoperative complications in CD patients undergoing abdominal surgery” (12). Instead, the recommendation remains to reduce the use of steroids as much as possible, as the use of steroids preoperatively has also been shown to increase the risk of postoperative septic complications, delay wound healing, and increase readmission rate (23). In our series, patients 1 and 2 discontinued their biological therapies at least 4 weeks before surgery without experiencing a disease flare-up. In patients 3 and 4, no biological therapies were in place, and nutritional intervention was conducted without the need for any pharmacological support, including steroids. In particular, in patient 4, it was possible to postpone surgery for up to 8 weeks, without concomitant pharmacological therapy, while maintaining reasonable disease control.

These observations align with the existing evidence regarding the efficacy of CDED plus PEN in maintaining clinical remission and an adequate nutritional status. None of our patients underwent surgery for hypoalbuminemia. Hypoalbuminemia is common in patients with active CD and is associated with increased postoperative complications even in patients with normal BMI (24). Maintaining adequate albumin levels suggests that luminal inflammation is under control, which is essential for reducing postoperative risk.

None of the patients experienced postoperative complications; however, it is worth emphasizing that the use of minimally invasive surgery may have contributed to a lower risk of complications (25).

In conclusion, the use of CDED+PEN in CD patients who are candidates for surgery was well tolerated and associated with good adherence. All patients demonstrated reasonable disease control without pharmacological therapy, and none experienced complications in the postoperative period. These observations support the implementation of CDED+PEN in the prehabilitation protocol, tailored explicitly for CD patients, even in the absence of evident malnutrition. Further studies are currently advocated to investigate the efficacy of CDED in optimizing surgical outcomes and preventing complications in CD patients undergoing surgery.

## Data Availability

The original contributions presented in the study are included in the article/supplementary material, further inquiries can be directed to the corresponding author.
